# Third‐degree atrioventricular block caused by intoxication with rhododendron leaves

**DOI:** 10.1111/anec.13012

**Published:** 2022-10-18

**Authors:** Liu Jiang, Lin Zhanxiong, Yao Fengcai, Liang Fuli, Su Xiaoling

**Affiliations:** ^1^ Department of Cardiac Function, Xi'an No. 3 Hospital The Affiliated Hospital of Northwest University Xi'an China; ^2^ Department of Cardiovascular Medicine Datong County People's Hospital Xining China; ^3^ Department of Cardiovascular Medicine Qinghai Provincial People's Hospital Xining China; ^4^ Department of Cardiovascular Medicine Qinghai Cardio‐Cerebrovascular Specialty Hospital, Qinghai High Altitude Medical Research Institute Xining China

**Keywords:** bradycardia, grayanotoxin, hypotension, *rhododendron*, third‐degree atrioventricular block

## Abstract

We report the case of a 41‐year‐old human with third‐degree atrioventricular block caused due to intoxication with water concoction prepared from *Rhododendron* leaves. Such poisoning is rare. It is prone to arrhythmia with hemodynamic instability and is confused with various diseases. For these reasons, the correct diagnosis and treatment of this poisoning are particularly important. We confirmed it by analyzing the remaining liquid carried by the family members. After symptomatic and supportive treatment, the patient was discharged uneventfully.

## Graphical abstract

The dreaded *Rhododendron* flowers and leaves: reversible third‐degree atrioventricular block.

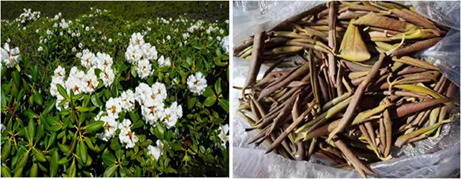

*Rhododendron* is a large genus of plants that are mainly found in the temperate and subtropical countries such as Turkey, Spain, Portugal, and Nepal, and China harbors 60% of the world's species. Qinghai Province is located in the northwest China. It is a high‐altitude area with relatively concentrated biodiversity and is an important haven for species' gene pool on the Qinghai–Tibet Plateau. *Rhododendron qinghaiense* mainly grows on the shady slopes of the mountains at an altitude of 4300 m. *Rhododendron* plants contain veratrotoxin, a poisonous component that can damage the heart and nervous system. The toxic effect of veratrotoxin on cardiomyocytes is mainly imparted through the sodium channel that keeps the cells in a depolarized state (Jansen et al., 2012).

Previous studies have shown that consuming azalea‐based honey can lead to symptoms of poisoning such as bradycardia, hypotension, nausea, and vomiting; however, no study has shown that azalea leaves can also cause acute poisoning symptoms. Herein, we report a case of a third‐degree atrioventricular block caused due to azalea leaf poisoning. To the best of our knowledge, this is the first case of acute poisoning caused due to azalea leaf consumption in our region, and we hope to draw attention to food poisoning caused by azalea leaves and its secondary products.

## 1 CASE PRESENTATION

**FIGURE 1:**
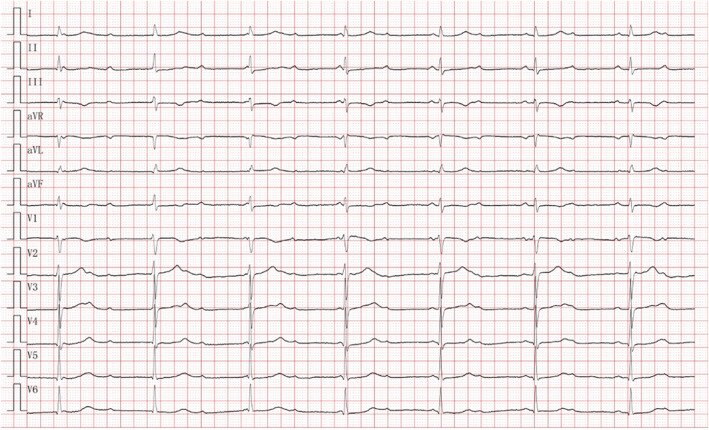
Third‐degree atrioventricular block

A 41‐year‐old human presented with blurred vision, dizziness, nausea, and vomiting. Physical examination on admission revealed a body temperature, blood pressure, ventricular rate, and respiration rate of 35.5°C, 60/40 mmHg, 42 beats/min, and 24 breaths/min, respectively. He exhibited profuse sweating; cold, clammy skin; limb weakness; mental fog; moderate face and lip cyanosis; and poor circulation in the extremities. The lungs were clear on auscultation without abnormal breath sounds, and the heart sounds were weak and regular with no pathological murmurs in any valve auscultation area. Emergency electrocardiography revealed a third‐degree atrioventricular block (Figure 1). In the serum biochemical examination, the potassium level was 3.26 (3.5–5.5) mmol/L; the results for routine blood and urine examination, liver and kidney function, and myocardial enzymes were normal. The patient had lived for a long time in the high‐altitude areas of the Qinghai Province and did not have a family history of any hereditary disease.

Upon further inquiry, the patient's family informed that he had a history of hypertension, for which oral antihypertensive drugs were ineffective. He was previously informed that a *Rhododendron* leaf water decoction could lower blood pressure; the aforementioned symptoms occurred within 30 min after ingestion. The residual liquid was tested for toxins and was confirmed to contain grayanotoxin (GTX). Symptomatic and supportive treatment such as fluid replacement and myocardial nutrition management were administered. The symptoms relieved after approximately 8 h, and no complications were observed after 24 h. After discharge and during 3 months of follow‐up, no discomfort was reported, and no abnormalities were found in electrocardiographic re‐examination.

## 2 DISCUSSION

**FIGURE 2:**
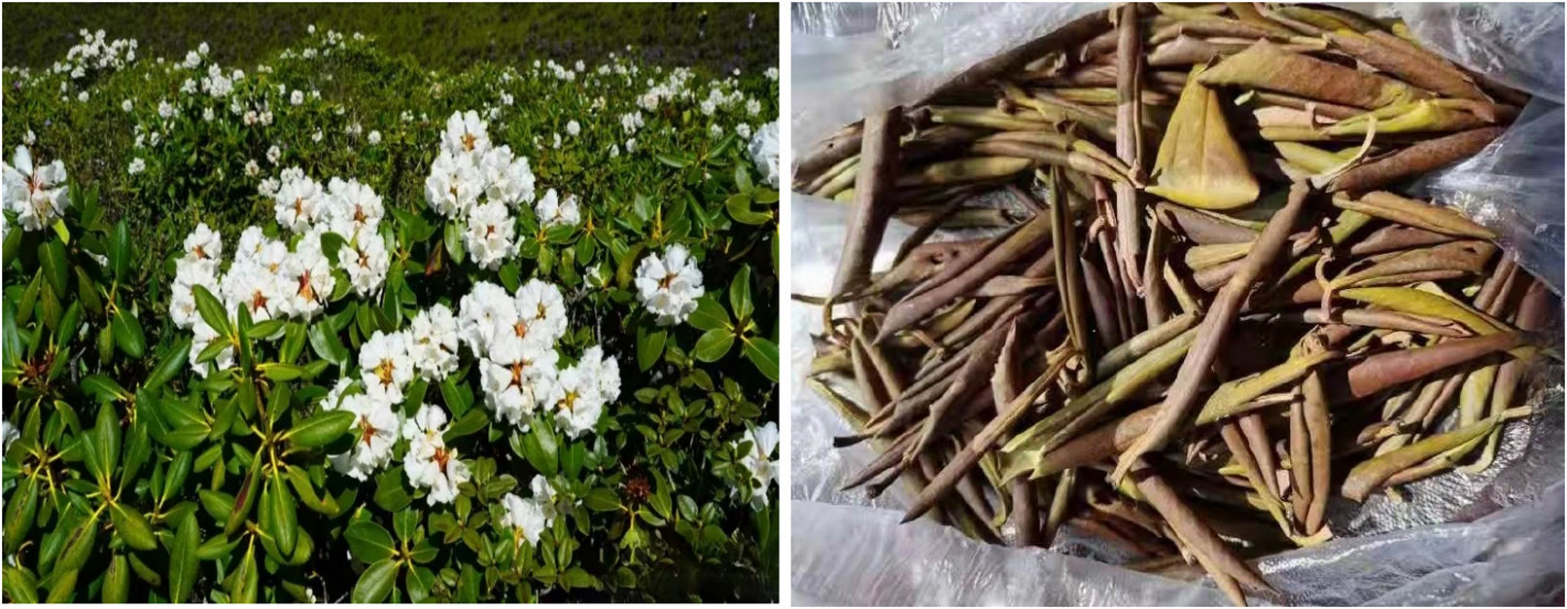
*Rhododendron* flowers and leaves

The Ericaceae family includes 127 genera that are found particularly in the temperate zones of the Southern and Northern Hemispheres and the subtropical zones of the Northern Hemisphere. Twenty‐two genera (±1065 species) are distributed across China, and many woody plant species are distributed in the western region due to the climatic conditions, favorable environment, and rich forest plant resources. The inhabitants of Qinghai Province refer to rhododendrons with large leaves and tall branches as Dongqing (Figure 2). Rhododendrons have various medicinal properties and are used to treat diseases such as hypertension, diabetes, cardiovascular diseases, rheumatic diseases, and diseases of the digestive system (Jansen et al., 2012). However, they also contain a toxic component, GTX, that can damage the heart and nervous system.

GTXs are polyhydroxylated cyclic hydrocarbons without nitrogen and are mainly divided into three subtypes. GTX‐I is the main subtype associated with cardiac manifestations. GTX‐II can suppress impulses from the sinoatrial node and may cause GTX‐II‐induced depolarization (Nakao & Seyama, 1984). Lastly, GTX‐III can elicit arrhythmias, which has been confirmed in animal studies; the possible underlying mechanism is related to the triggered activity in oscillatory form after inducing potential (Brown et al., 1981). The main targets of GTXs are skeletal muscle, cardiac muscle, and central and peripheral neurons (Onat et al., 1991). They stimulate the opening of sodium channels in the cell membrane, which depolarize and excite cells, leading to the activation of the vagus nerve (Jansen et al., 2012). Among all types of GTXs, GTX‐I not only affects the sinoatrial node but also affects the conduction of the atrioventricular node, and this might be the underlying reason of the third‐degree atrioventricular block in our patient.

The keywords “grayanotoxin” and “atrioventricular block” were used to retrieve articles published between January 2006 and May 2022 from PubMed; only three case reports regarding third‐degree atrioventricular block due to the consumption of poisonous honey (mainly produced by rhododendrons) were retrieved. A retrospective study by Demir et al. (2011) reported that 100% of patients who consumed honey‐containing GTX exhibited dizziness and weakness; 85% exhibited excessive sweating, nausea, and vomiting; and 66% exhibited common symptoms, such as hypotension. In their study, the mean pulse rate, systolic blood pressure, and duration of hospital stay were 56 beats/min, 102 mmHg, and 14.7 h, respectively. Sinus rhythm, sinus bradycardia, nodal rhythm, and atrial fibrillation accounted for 48%, 33%, 14%, and 5% of symptoms, respectively. Atropine was administered to 86% of the patients, while 14% patients did not require atropine. No deaths were reported, and all patients were discharged without any complications.

A prospective study by Okuyan et al. (2010) reported that most patients experienced nausea, vomiting, dizziness, fainting, and sweating after consuming toxic honey. Among their cohort, one and five patients presented with generalized epileptic seizure and syncope, respectively. The mean systolic blood pressure, diastolic blood pressure, and heart rate fluctuated between 50 and 100 mmHg, 40 and 70 mmHg, and 25 and 59 beats/min, respectively, and the duration of hospital stay ranged from 12 to 36 h. Sinus bradycardia, complete atrioventricular block, and nodal rhythm occurred in 43%, 36%, and 21% of patients, respectively, and 75% patients responded favorably to atropine (0.5–1 mg) without any complications.

In conclusion, literature reports suggest that most patients consumed toxic honey from the Black Sea Region of Turkey, which caused common symptoms (hypotension, nausea, vomiting, and dizziness); in severe cases, syncope, atrioventricular block, myocardial infarction, blurred vision, and epilepsy may occur. Symptoms of the patients were relieved within 24 h after treatment (including atropine and fluid administration) with a good prognosis; however, a few deaths were reported.

Therefore, clinicians should consider certain species of *Rhododendron*, whose leaves and secondary products contain GTXs that may lead to acute poisoning, causing hypotension, bradycardia, dizziness, and abdominal discomfort. Detailed information regarding the patients' current, past, and dietary history is essential for an accurate diagnosis. In cases of unexplained hypotension, nausea, vomiting, and atrioventricular block, GTX poisoning should be considered for a differential diagnosis. Additionally, prompt and effective treatment should be administered to patients with severe conditions to avoid misdiagnosis and unnecessary medical treatment.

## AUTHOR CONTRIBUTIONS

LJ, LZX, and YFC collected medical records. LJ and LFL wrote the manuscript. SXL reviewed the manuscript. All authors read and approved the final manuscript. All authors agreed to their contributions.

## CONFLICT OF INTEREST

The authors have explicitly stated that there are no conflicts of interest in connection with this article.

## ETHICAL APPROVAL

Written informed consent was obtained from the patient described in this case report.

## PATIENT CONSENT STATEMENT

The patient has consented to the publication.

## PERMISSION TO REPRODUCE MATERIAL FROM OTHER SOURCES

Not applicable.

## Data Availability

Data sharing is not applicable to this article as no new data were created or analyzed in this study.
